# Neuroprotective effects of rutin against cuprizone-induced multiple sclerosis in mice

**DOI:** 10.1007/s10787-024-01442-x

**Published:** 2024-03-21

**Authors:** Mariam A. Nicola, Abdelraheim H. Attaai, Mahmoud H. Abdel-Raheem, Anber F. Mohammed, Yasmin F. Abu-Elhassan

**Affiliations:** 1 Department of Pharmacology and Toxicology, Faculty of Pharmacy, Assiut University, Asyût, 71526 Egypt; 2 Department of Anatomy and Histology, School of Veterinary Medicine, Badr University in Assiut, New Nasser City, West of Assiut, Asyût, Egypt; 3 Department of Anatomy and Embryology, Faculty of Veterinary Medicine, Assiut University, Asyût, 71526 Egypt; 4 Department of Pharmacology, Faculty of Medicine, Assiut University, Asyût, Egypt; 5 Department of Pharmaceutical Organic Chemistry, Faculty of Pharmacy, Assiut University, Asyût, 71526 Egypt

**Keywords:** Multiple sclerosis, Cuprizone, Rutin, Antioxidant, Anti-inflammatory

## Abstract

Multiple sclerosis (MS) is a chronic inflammatory neurodegenerative disease of the central nervous system that injures the myelin sheath, provoking progressive axonal degeneration and functional impairments. No efficient therapy is available at present to combat such insults, and hence, novel safe and effective alternatives for MS therapy are extremely required. Rutin (RUT) is a flavonoid that exhibits antioxidant, anti-inflammatory, and neuroprotective effects in several brain injuries. The present study evaluated the potential beneficial effects of two doses of RUT in a model of pattern-III lesion of MS, in comparison to the conventional standard drug; dimethyl fumarate (DMF). Demyelination was induced in in male adult C57BL/6 mice by dietary 0.2% (w/w) cuprizone (CPZ) feeding for 6 consecutive weeks. Treated groups received either oral RUT (50 or 100 mg/kg) or DMF (15 mg/kg), along with CPZ feeding, for 6 consecutive weeks. Mice were then tested for behavioral changes, followed by biochemical analyses and histological examinations of the corpus callosum (CC). Results revealed that CPZ caused motor dysfunction, demyelination, and glial activation in demyelinated lesions, as well as significant oxidative stress, and proinflammatory cytokine elevation. Six weeks of RUT treatment significantly improved locomotor activity and motor coordination. Moreover, RUT considerably improved remyelination in the CC of CPZ + RUT-treated mice, as revealed by luxol fast blue staining and transmission electron microscopy. Rutin also significantly attenuated CPZ-induced oxidative stress and inflammation in the CC of tested animals. The effect of RUT100 was obviously more marked than either that of DMF, regarding most of the tested parameters, or even its smaller tested dose. In silico docking revealed that RUT binds tightly within NF-κB at the binding site of the protein-DNA complex, with a good negative *score* of −6.79 kcal/mol. Also, RUT-Kelch-like ECH-associated protein 1 (Keap1) model clarifies the possible inhibition of Keap1–Nrf2 protein–protein interaction. Findings of the current study provide evidence for the protective effect of RUT in CPZ-induced demyelination and behavioral dysfunction in mice, possibly by modulating NF-κB and Nrf2 signaling pathways. The present study may be one of the first to indicate a pro-remyelinating effect for RUT, which might represent a potential additive benefit in treating MS.

**Graphical Abstract:**

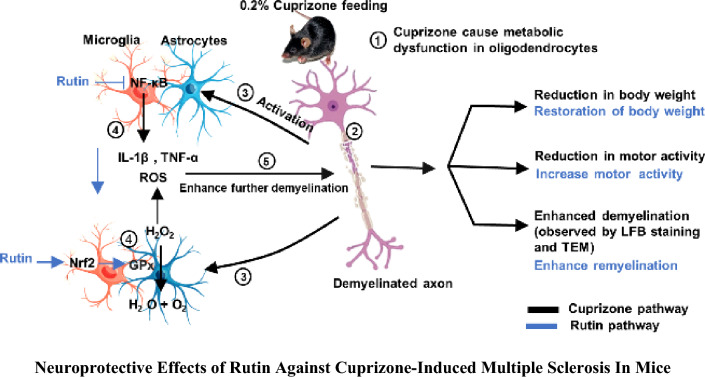

## Introduction

Multiple sclerosis is a chronic, inflammatory, progressive, demyelinating autoimmune disease that damages the myelin sheath in the central nervous system (CNS), resulting in several neurological impairments (Titus et al. 2022). These include variable changes, from cognitive and psychological dysfunction to sensory and motor impairments (Sen et al. 2020, 2022). It appears that there is a causal relationship between environmental and genetic factors (Dobson and Giovannoni 2019). This devastating condition mainly affects people between the ages of 20 and 40 (Klineova and Lublin 2018), and is regarded as the main cause of disability of neurological origin among young adults (Krokavcova et al. 2008).

Despite the lack of knowledge regarding aetiopathogenesis, it is believed that MS is an inflammatory autoimmune disease. Reactive lymphocytes that cross the blood–brain barrier (BBB), and then become activated, are responsible for the pathogenic mechanisms underlying the CNS lesions in MS. Oxidative stress, cytotoxicity, axonal damage, astrogliosis, and microgliosis are all consequences of the persistent neuroinflammation that the adaptive immune response creates (Pérez-Cerdá et al. 2016; Abdel-Maged et al. 2020). However, aside from the effect on the white matter constituted by myelin, the gray matter is also affected, causing major cortical changes and tissue loss, and leading to varying degrees of impairment in cognitive functions (Geurts and Barkhof 2008).

In the early stages of MS, the remyelination process can protect the myelin sheath due to the existence of mature oligodendrocytes (OLs) and the removal of debris by microglia. In late stages, however, the remyelination process decreases and becomes overcome as the pathophysiological processes accountable for demyelination eventually predominate, resulting in a significant loss of myelin sheath. Initially, this is linked with a decrease in action potential conduction through axons, and later on in advanced cases, the action potential is completely inhibited, ensuing loss of signals between the CNS and peripheral organs (Nakahara 2017; Sen et al. 2022).

Current MS treatment plans focus on reducing the immune response, with ongoing efforts to treat the disease underlying pathology or promote the healing of the injured tissues (Coles et al. 2022; Filippi et al. 2022). Nonetheless, these therapies are only moderately effective in treating MS, and primarily, they have variable effects on the degenerative components of the disease, with high costs and unfavorable safety profiles. Patients with MS may become tolerant to these medications or experience serious adverse effects such as progressive multifocal leukoencephalopathy (Hauser and Cree 2020). Consequently, there is a constant need for new medications with improved safety profiles and novel mechanisms of action. Finding an alternate MS therapeutic strategy is crucial to enable the repair of damaged myelin and preventing neurodegeneration. Additionally, the idea of drug repurposing is embraced to be the most recent technique in drug research because of its effectiveness and economic impact.

Accumulating evidence suggests that oxidative stress and inflammation contribute to the etiology of MS. Inflammation stimulates microglial and astrocyte activity, which leads to their recruitment and release of pro-inflammatory cytokines (e.g., tumor necrosis factor-alpha; TNF-α, interleukin-1β; IL-1β and interferon-γ; IFN-γ) that trigger the demyelination process (Voss et al. 2012; El Sharouny et al. 2021). Notably, nuclear factor κB (NF-κB) is one of the master transcription factors that control inflammation through enhancement of the expression of a variety of inflammatory mediators (Yue et al. 2018).

Meanwhile, oxidative stress is a key factor resulting in OLs damage and axonal harm (Draheim et al. 2016). Reactive oxygen and nitrogen species, which cause demyelination and axon disruption in MS, are primarily created by activated macrophages and microglia (Cherry et al. 2014; El Sharouny et al. 2021). Importantly, the transcription factor (erythroid-derived) nuclear factor-like 2 (Nrf2) is a crucial antioxidative defense mechanism that regulates the expression of antioxidant proteins and shields cells from oxidative stress during inflammation and damage. In light of that, compounds with antioxidant and anti-inflammatory activities could be superior to many other available MS treatments (Carlson and Rose 2006).

Specifically, natural polyphenolic compounds from medicinal plants have received considerable interest, on account of their antioxidant, anti-inflammatory and neuroprotective properties, that now represent promising targets for MS (Benlloch et al. 2020). Among these compounds, flavonoids; a group of compounds with diverse phenolic structures, are important natural products found in different fruits and vegetables (Kuzu et al. 2018). Rutin (quercetin-3-rutinoside; RUT), also called rutoside or sophorine, is a citrus flavonoid found in variety of foods including apples, onions, red wine, and tea (Ola et al. 2015). It has a wide variety of biological and pharmacological properties, such as anti-inflammatory, antioxidant, antihypertensive, anti-apoptotic, and anticancer activities (Perk et al. 2014). Additionally, RUT was reported to cross the BBB (Anjomshoa et al. 2020), and defend against a number of CNS injuries through antioxidant/anti-inflammatory mechanisms. Yet, to our knowledge, no reports have so far indicated the potential benefit of RUT in MS. Consequently, the present study was designed to estimate the possible neuroprotective effects of two doses of RUT against cuprizone (CPZ)-induced demyelination in mice, in comparison to a conventional standard drug; dimethyl fumarate (DMF). Also, a trial was carried out to investigate the underlying mechanisms mediating these effects, by following the effect of treatment on inflammatory and oxidative stress markers; histopathological changes, as well as molecular docking with target transcription factors.

## Materials and methods

### Animals

Thirty male C57BL/6 mice (6–8 weeks old), weighing 20–25 g, were purchased from the Theodor Bilharz Research Institute (Giza, Egypt) and housed in the Assiut University animal house facility. Mice were given a week to acclimate before the start of the experiment. Mice were then kept in groups of 6 per cage in controlled environmental conditions at a constant temperature (25 ± 2 °C) and under a 12/12-h light/dark cycle. Animals were weighed independently at the commencement of the study, every week, and before sacrifice. Mice were provided access to a standard chow diet and water ad libitum. All experiments were performed according to protocols approved by the Institutional Animal Care and Use Committee guidelines of the Faculty of Medicine, Assiut University (Approval No. 17300777).

### Chemicals

Cuprizone [bis(cyclohexanone) oxaldihydrazone] was purchased from Sigma-Aldrich Co. (USA). Rutin was obtained from Acros Organics (Belgium). Dimethyl fumarate was purchased from Hikma (Egypt). Spectrophotometric kits for glutathione peroxidase (GPx) and malondialdehyde (MDA) were purchased from Biodiagnostic (Giza, Egypt). Mouse TNF-α and IL-1β ELISA kits were purchased from MyBioSource (San Diego, USA) and Sunred (Shanghai; China), respectively. All other chemicals were of the highest analytical grade and purchased from Sigma-Aldrich Co. (USA) or Merck (Germany).

### Experimental design

#### Induction of multiple sclerosis

Multiple sclerosis was induced in mice using CPZ. C57Bl/6 mice were fed chow containing 0.2% (w/w) of CPZ, for 6 consecutive weeks (Largani et al. 2019; Tomas-Roig et al. 2020). Cuprizone was freshly prepared each day over the 6 weeks of the experiment.

#### Animal groups

Animals were divided into five groups (*n* = 6). Group I consisted of healthy mice fed with normal chow for 6 weeks (normal control; NC). Group II mice were fed a diet containing 0.2% (w/w) CPZ mixed with standard rodent chow for 6 weeks (Cuprizone group; CPZ). Group III mice were fed on CPZ, and simultaneously received 15 mg/kg DMF (Schilling et al. 2006; Linker et al. 2011), twice daily orally by gavage, as a standard drug (CPZ + DMF). Group IV mice were fed on CPZ, and simultaneously received rutin (50 mg/kg; p.o; CPZ + RUT50) (Ramalingayya et al. 2016; Çelik et al. 2020). Group V mice were fed on CPZ concurrently with an oral dose of 100 mg/kg of rutin (CPZ + RUT100) (Ramalingayya et al. 2016; Çelik et al. 2020). Oral administration of either the standard drug or RUT was performed daily for 6 successive weeks (the experimental period).

#### Body weight recording

To estimate the effect of 0.2% CPZ feeding and treatments on weight changes of C57BL/6 mice, the body weight of each experimental animal was recorded weakly throughout the course of the experiment. Also, the final body weight, just before the animal killing, was recorded.

### Determination of the effect of treatment on cuprizone-induced demyelination

#### Behavioral and motor studies

Animals were behaviorally tested in the last 3 days of the treatment protocol for motor coordination and equilibrium, with a minimum of 30 min between each trial. All tests were carried out during the animal’s light cycle to reduce any potential circadian fluctuation.

#### Rotarod test

Studies have shown that CPZ, in addition to the CC which is the most affected brain region, also induces demyelination in other motor regions in the brain, including the cerebellum and basal ganglia (Pott et al. 2009; Goldberg et al. 2015). Animal balance and motor coordination were tested using a rotating metal rod that is 3 cm in diameter and 30 cm high, rotating at a speed of 25 rpm. Before experimentation, mice were acclimated on the rotarod via 3 training sessions, lasting 5 min each, for 3 days. The latency to fall off the rotarod within 5 min for each animal was recorded (Elbaz et al. 2018).

#### Locomotor activity (actophotometer test)

Actophotometer was used to measure the locomotor activity. The photocell was hit by a laser beam that was cut off by the movement of the animal, and the counts were recorded and displayed digitally. Individually positioned mice were placed within the apparatus, and the activity count was recorded for 5 min. The locomotor activity was quantified as total photobeam counts/5 min (Garabadu and Agrawal 2020).

#### String test for grip strength

A technique for evaluating grip strength that considers neuromuscular activity is the string test (Takeshita et al. 2017). Mice were permitted to hang, by their forelimbs, a horizontal steel wire that was 35 cm long and 2 mm in diameter and had been extended horizontally with a 50 cm height. The time spent by each mouse hanging on the wire before slipping off was recorded, with a cut-off value of 3 min (Abd El Aziz et al. 2021; Ammar et al. 2022).

#### Open-field test (OFT)

The OFT was performed in a square wooden box measuring 40 × 40 × 25 cm. The box has red walls and a black floor that was divided into a 4 × 4 grid with 16 equal squares by white lines. Each mouse was carefully placed in the center of the wooden box, and the locomotor activity was videotaped for 3 min. Before and after each trial, the equipment was cleaned with 70% ethanol to get rid of any remaining odors or other debris. To eliminate any outside cues, the test was conducted in a quiet space with white light. The immobility time (the duration of time the animal was immobile during testing), rearing frequency (the number of times it stood on its hind limbs without forelimbs), and ambulation frequency (the number of squares it traversed) were all calculated for each animal (Ohl et al. 2016; Abd El Aziz et al. 2021).

### Specimen collection

The biggest white matter structure in the brain is the CC, which connects the homologous cortical regions of the two cerebral hemispheres and is essential for the transmission of sensory, cognitive, and motor information. Biochemical and histological examinations of the present study thus focused on the CC; one of the most extensively researched regions in the CPZ demyelination model.

At the end of the behavioral tests, animals were weighed, deeply anesthetized with ketamine, and transcardially perfused with cold phosphate-buffered saline (PBS). The whole brains were then isolated and weighed. Brain tissue of each mouse was bisected into two hemispheres; the right ones were immediately dissected on ice, and the CC was abstracted, weighed, and stored at −80 °C to be used for biochemical analyses. Upon testing, tissues were homogenized in PBS with an electric homogenizer and then centrifuged at 10,000 rpm for 20 min at 4 °C. Placed on ice, aliquots of the supernatants were transferred to fresh, chilled tubes and stored at −80 °C. The left hemispheres were again bisected transversely at the level of the striatum into two portions. The caudal one was fixed for 48 h in 10% neutral-buffered formalin to be used for histopathological studies; the other was fixed with 2% glutaraldehyde for electron microscope examination.

### Histopathological studies

To analyze the loss of myelin in the CC of CPZ-fed mice, together with the effect of different treatments, luxol fast blue/cresyl-violet (LFB/CV) staining and transmission electron microscopy (TEM) were performed.

#### Luxol fast blue/cresyl-violet staining

Luxol fast blue/cresyl-violet staining was used to assess myelinated nerve fibers in the CC histochemically (Kuhlmann et al. 2017). Tissue blocks were made for paraffin cutting. Briefly, escalating degrees of ethyl alcohol were used to dehydrate fixed tissues. Samples were cleared by first dipping them in xylene twice and then embedding them in paraffin wax (Sigma-Aldrich, USA). Step serial sagittal sections (10 sections per animal) from the CC were cut at 5 µm thickness by Richert Leica RM 2125 Microtome (Germany) and fit on glass slides. Sections were then stained with LFB/CV. Fleetingly, sections were immersed in 0.1% LFB solution at 56 °C overnight, before being rinsed in ethanol and distilled water (dH2O) to remove any remaining blue stain. Afterward, sections were differentiated by quick immersion in lithium carbonate solution (0.01%) and washed thoroughly in distilled water, then counterstained with CV for 10 min to deepen the color of LFB-stained myelin from turquoise to   deep blue. Subsequently, sections were rinsed successively in water, dehydrated, cleared in xylene, and mounted with nonaqueous dibutylphthalate polystyrene xylene (DPX) (Bancroft and Gamble 2008; Attaai et al. 2020). After being allowed to dry overnight, the slides were examined under an Olympus CX41 light microscope equipped with a digital Olympus camera (C-5060, Japan), and processed using Image J software at 20×  magnification, and the percentage of myelination was measured.

#### Transmission electron microscopy

The status of myelin can also be assessed using TEM (Marzan et al. 2021). The staining method was carried out according to Suvarna et al. (2018) and Abd-Elhafeez et al. (2021). The specimens that were soaked in a 2% glutaraldehyde fixative solution were incubated overnight. Following fixation, samples were washed in phosphate buffer before being osmicated at a pH of 7.3 with 1 percent osmium tetroxide in 0.1 mol/L Na-phosphate buffer. After that, samples were dehydrated in a series of ethanol, propylene oxide, and Araldite embeddings. Using a Reichert Ultratome (Leica, Germany), semi-thin slices were cut at 1 μm thickness and stained with toluidine blue for light microscopy to select the region of interest for TEM. At Assiut University Electron Microscopy Unit, Ultrotom VRV was used for ultrathin sectioning (LKB Bromma, Germany). The slices (70 nm) were stained with uranyl acetate and lead citrate before being examined in a JEOL 100CX II TEM (JEOL, Tokyo, Japan). According to the procedures outlined in prior studies, the number of myelinated fibers (on at least 150 axons/animal) and the G-ratio (the ratio of axonal diameter to fiber diameter) were calculated. For each myelinated fiber, the G-ratio was determined, and then the average was calculated for all the G-ratio values from one brain. The mean of the average G-ratio from the three brain samples was determined for each group (Lombardi et al. 2019; Tahmasebi et al. 2021). ImageJ software was used, and quantifications were performed on three mice per group.

### Biochemical parameters

#### Measurement of the level of proinflammatory cytokines in the CC

To ascertain the role that proinflammatory cytokines play in CPZ-induced MS, and to evaluate how therapy could affect neuroinflammation characterizing MS, homogenates were used for the determination of TNF-α and IL-1β using ELISA kits. The assay was implemented according to the manufacturer’s instructions. Results of TNF-α and IL-1β were presented as ng/g and pg/g of tissue, respectively.

#### Measurement of the CC level of oxidant/antioxidant markers

As an expected mechanism for the enhancement of remyelination in animals, the oxidant/antioxidant balance in the CC of CPZ-fed mice was followed. Homogenates were used for evaluating the lipid peroxidation indicator; MDA and results were expressed as nmol/g tissue. Also, the activity of the antioxidant biomarker; GPx, was assessed, and results were expressed as U/g tissue. The assays were performed colorimetrically following the manufacturers’ guidelines.

### Molecular modeling

All modeling calculations and docking studies were done on a Processor Intel(R) Pentium(R) CPU N3510@ 1.99 GHz and 4 GB Memory with Microsoft Windows 8.1 pro (64 Bit) operating system using Molecular Operating Environment (MOE 2019.0102, 2020; Chemical Computing Group, Canada) as the computational software. Rut structure was retrieved from the PubChem database as an STD file, then visualized, prepared, and minimized via MOE interface. All MOE minimizations were done until a RMSD gradient of 0.01 kcal/mol/Å using (OPLS-AA) force field to calculate the partial charges automatically using Distance solvation. DNA and other irrelevant ligands were removed. Before simulations, the protein was protonated using Quick Prep function. Initial placement was achieved using Triangle matching with London dG scoring, and then the top 30 poses were refined using force field (OPLS-AA) and GBVI/WSA dG scoring. The output dock database file was created with 5 poses for each ligand and arranged according to the final score function (S).

### Western blot

The ReadyPrepTM protein extraction kit (total protein), provided by Bio-Rad Inc. (Catalog #163-2086), and employed according to manufacturer instructions, was added to each sample of the homogenized tissues of different groups. Bradford Protein Assay Kit (SK3041) for quantitative protein analysis was provided by Bio basic Inc (Markham Ontario L3R 8T4 Canada). A Bradford assay was performed according to manufacturer protocols to determine protein concentration in each sample. 20 μg protein concentration of each sample was then loaded with an equal volume of 2× Laemmli sample buffer containing 4% SDS, 10% 2-mercaptoehtanol, 20% glycerol, 0.004% bromophenol blue and 0.125 M Tris HCl. The pH was checked and brought to 6.8. Each of the previous mixtures was heated at 95 °C for 5 min prior to loading on polyacrylamide gel electrophoresis to accomplish denaturation of the protein. The gel was assembled in transfer sandwich as following from below to above. The sandwich was put into the transfer tank with the 1× transfer buffer, which contains 25 mM Tris, 190 mM glycine, and 20% methanol. The BioRad Trans-Blot Turbo was then used to transfer protein bands from gel to membrane for 7 min at 25 V. The membrane was blocked for 1 h at room temperature in Tris-buffered saline with Tween 20 (TBST) buffer and 3% bovine serum albumin (BSA). The components of blocking buffer were as follows: 20 mM Tris, pH 7.5, 150 mM NaCl, 0.1% Tween 20 and 3% bovine serum albumin (BSA). The primary antibodies; mouse monoclonal Nrf2 antibody (1:400, SANTA CRUZ BIOTECHNOOLOGY, INC) and β-actin, were diluted in TBST according to manufacturer instructions. Each primary antibody solution was incubated against the blotted target protein for an entire night at 4 °C. The blot was rinsed 3–5 times for 5 min with TBST. The target protein was incubated in the HRP-conjugated secondary antibody solution (Goat anti-rabbit IgG-HRP-1 mg Goat mab—Novus Biologicals) for 1 h at room temperature. With TBST, the blot was rinsed 3–5 times for 5 min.

The chemiluminescent substrate (Clarity TM Western ECL substrate Bio-Rad cat#170-5060) was applied to the blot according to the manufacturer’s recommendation. An imager with a CCD camera was used to record the chemiluminescent signals. The band intensity of the target proteins was read against the beta actin of the control sample using image analysis software (housekeeping protein) by protein normalization on the ChemiDoc MP imager.

### Statistical analysis

Results of the current study were analyzed using Graph Pad Prism 8 (GraphPad Software, Inc.) and presented as mean ± standard error of the mean (*M* ± SEM). Comparisons among different groups were performed using one-way analysis of variance (ANOVA) followed by Tukey’s post hoc test. For all statistical comparisons, a *p* value <0.05 was considered statistically significant.

## Results

### Effect of rutin on cuprizone-induced weight loss

**Fig. 1:**
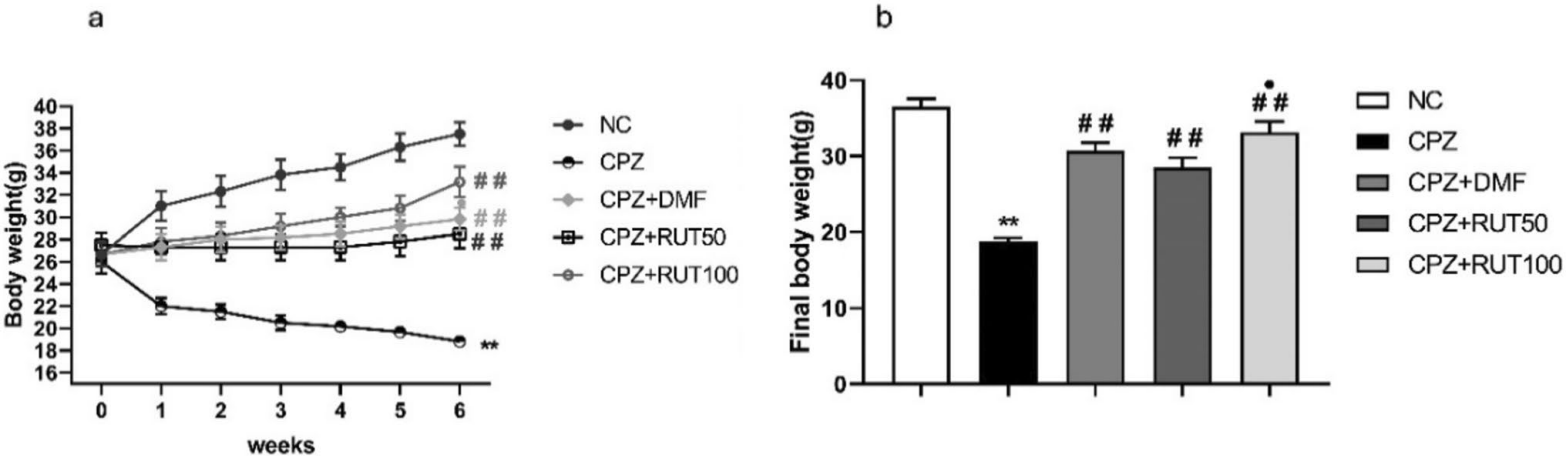
Amelioration of body weight loss by RUT in CPZ-induced demyelination in mice throughout the 6 weeks of the experiment. **a** The average body weight change in the NC, CPZ, CPZ + DMF, CPZ + RUT50, and CPZ + RUT100 groups during the 6-week-experimental period. **b** The final body weight of mice of different groups. Data are presented as mean ± SEM, *n* = 6 per group. ** *p*  < 0.01 vs. NC, ^##^ *p*  < 0.01 vs. CPZ, and ^•^ *p*  > *0.05* vs. CPZ + RUT50 (ANOVA). NC: control group fed on normal diet; CPZ: cuprizone group fed on a diet with 0.2% (w/w) cuprizone; DMF: dimethyl fumarate 15 mg/kg; RUT50: rutin 50 mg/kg; RUT100: rutin 100 mg/kg

Mice fed a normal diet showed a gradual increase in the average body weight during the six weeks. On the other hand, the body weight dramatically decreased in the CPZ-fed mice during the first week of intoxication (*p* < 0.01), while in the following 5 weeks, a slow reduction in the body weight was observed. Over the 6 weeks following induction, there was a clear distinction in the average body weight between the control and CPZ groups (*p* < 0.01). Mice that co-administered DMF, RUT50, and RUT100 with CPZ showed significantly less weight loss during the 6 weeks of the experiment when compared to the CPZ group (*p* < 0.01; Fig. 1a).

The final body weight was separately recorded before animal killing. Cuprizone-fed mice showed a significant reduction in the final body weight compared to normal diet fed-mice (18.83 ± 0.401 vs. 36.5 ± 1.022 g;* p* < 0.01). In contrast, mice of the groups that received DMF, RUT50, and RUT100 along with CPZ remarkably retained the natural increase in body weight compared to CPZ-fed mice (30.67 ± 1.116, 28.5 ± 1.285 and 33.17 ± 1.376 vs. 18.83 ± 0.401 g, respectively;* p* < 0.01). A significant difference in the average weight between RUT50 and RUT100 mice was detected, with a higher weight found in the RUT100 group (*p* < 0.01; Fig. 1b).

### Effect of rutin on cuprizone-induced behavioral and motor abnormalities

#### Rotarod test

**Fig. 2:**
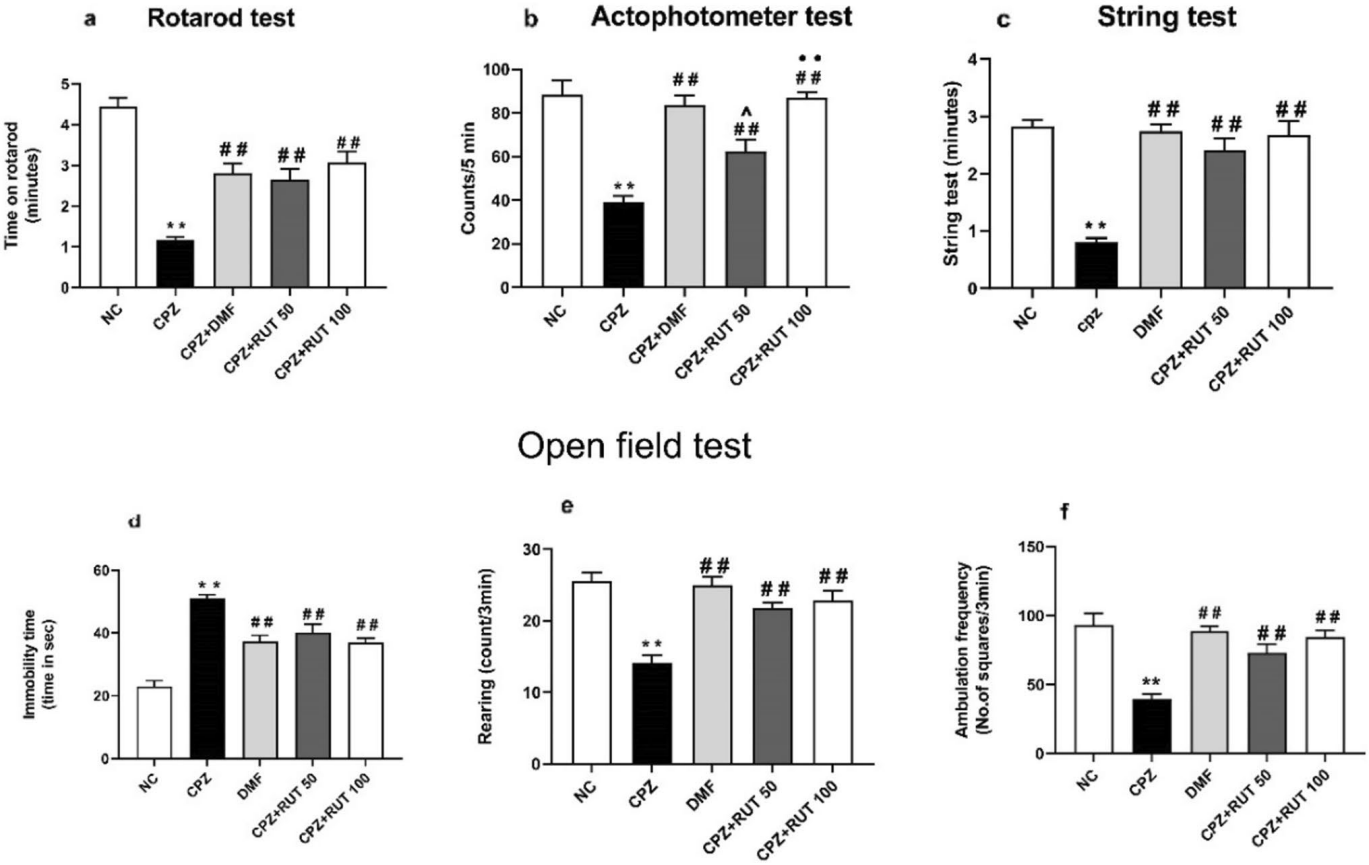
Effect of DMF and RUT treatments on neurobehavioral changes. **a** Rotarod test, **b** Actophotometer test, and **c** String test for grip strength. Open field test: **d** immobility time, **e** rearing, and **f** ambulation frequency. Data represent mean ± SEM (*n* = 6). ** *p*  < 0.01 vs. NC, ^##^ *p*  < 0.01 vs. CPZ group, ^^^ *p*  < 0.05 vs. CPZ + DMF, and ^••^ *p*  < 0.01 vs. CPZ + RUT50 (ANOVA). NC: control group fed on normal diet; CPZ: cuprizone group fed on diet enriched with 0.2% (w/w) cuprizone; DMF: dimethyl fumarate 15 mg/kg; RUT50: rutin 50 mg/kg; RUT100: rutin 100 mg/kg

Results showed that the time of staying on the rotating rod for the CPZ group was significantly lower as compared to the control group that received normal chow (1.171 ± 0.068 vs. 4.443 ± 0.216 min; *p* < 0.01). However, rotarod performance was markedly enhanced after DMF and RUT regimens (2.814 ± 0.224, 2.657 ± 0.279, and 3.071 ± 0.278 min for CPZ + DMF, CPZ + RUT50, and CPZ + RUT100, respectively; *p* < 0.01) in comparison to CPZ. Notably, there was no significant variation in the latency to fall among RUT50, RUT100, and DMF, or between RUT50 and RUT100 (Fig. 2a).

#### Locomotor activity (actophotometer test)

Results of the locomotor activity (Fig. 2b) clearly infer that CPZ caused substantial demyelination resulting in a significant reduction in locomotor activity compared to the negative control (39.14 ± 2.721 vs. 88.29 ± 6.593 counts/5 min; *p* < 0.01). However, there was a considerable increase in locomotor activity in DMF, RUT50, and RUT100 groups (83.71 ± 4.156, 62.57 ± 5.295, and 87.00 ± 2.400 counts/5 min, respectively; *p* < 0.01) compared to CPZ-fed mice. While there was no significant difference between the effects of RUT100 and the standard drug, DMF-treated animals showed markedly higher activity than RUT50 (*p* < 0.05). Also, RUT100 produced a more pronounced effect than RUT50 regarding this test (*p* < 0.01).

#### String test for grip strength

As shown in (Fig. 2c), mice of the CPZ group showed a significant decrease in the time that the animal took hanging on the steel wire compared to the control group (0.810 ± 0.072 vs. 2.833 ± 0.109 min; *p* < 0.01). On the other hand, DMF, RUT50, and RUT100 administration showed a remarkable increase in the grip strength compared to the CPZ group (2.741 ± 0.124 for CPZ + DMF, 2.407 ± 0.218 for CPZ + RUT50 and 2.670 ± 0.256 for CPZ + RUT100; *p* < 0.01). Herein, no significant difference was recorded among the treated groups regarding this test.

#### Open-field test

Compared to the negative control, mice treated with CPZ showed a clear increase in the immobility time (23.00 ± 1.844 vs. 51.17 ± 1.078 s; *p* < 0.01), as well as a reduction in rearing and ambulation frequency (25.50 ± 1.232 vs. 14.17 ± 1.078 count/3 min and 93.29 ± 8.234 vs. 39.57 ± 3.728 no. of squares/3 min; *p* < 0.01, respectively). Conversely, DMF, RUT50, and RUT100 mice showed a significant decrease in the immobility time compared to CPZ-intoxicated group (37.33 ± 1.909, 40.17 ± 2.600, 37.00 ± 1.438 vs. 51.17 ± 1.078 s, respectively; *p* < 0.01). Likewise, treated groups revealed a significant elevation in rearing and ambulation frequency compared to CPZ (CPZ + DMF, RUT50, and RUT100: 25.00 ± 1.125, 21.83 ± 0.749, 22.83 ± 1.352 vs. 14.17 ± 1.078 count/3 min for rearing record, and 88.71 ± 3.759, 73.00 ± 6.291, 84.71 ± 4.775 vs. 39.57 ± 3.723 no. of squares/3 min for ambulation frequency, respectively; *p* < 0.01, Fig. 2d–f).

### Effect of rutin on cuprizone-induced alterations in brain myelination status

**Fig. 3:**
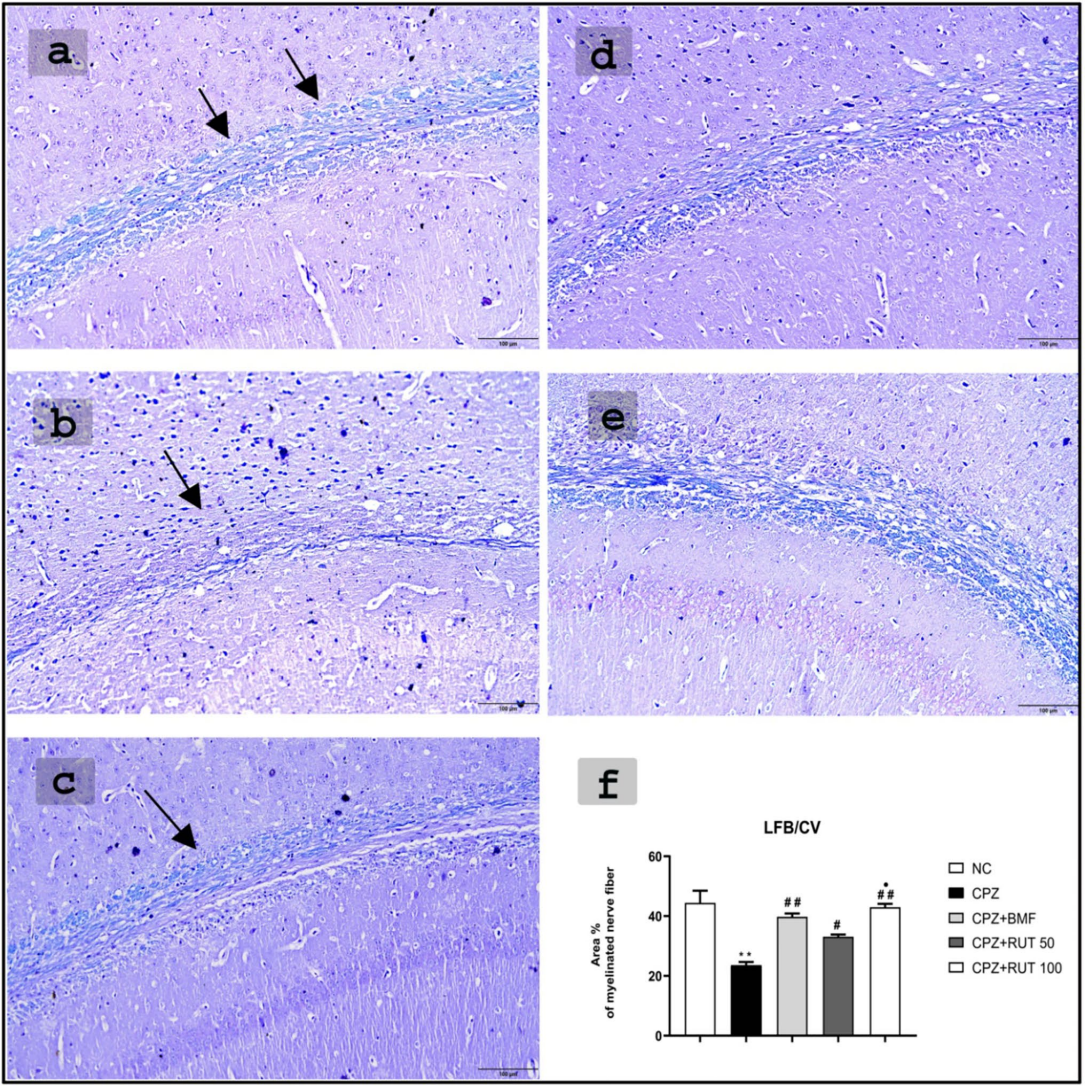
Evaluation of myelination in the CC by LFB/CV staining in C57BL/6 mice following 6 weeks of the experiment protocol in **a** NC; **b** CPZ; **c** CPZ + DMF; **d** CPZ + RUT50 and **e** CPZ + RUT100 groups. Demyelination was induced by CPZ, whereas DMF and RUT therapy abrogated CPZ-induced demyelination and restored the myelinated appearance of the CC. **f** Quantitative analysis of the percentage area of myelination in the CC by LFB/CV staining. ** *p*  < 0.01 vs. NC group, ^#^ *p*  < 0.05 and ^##^ *p*  < 0.01 vs. CPZ group, and ^•^ *p*  < 0.05 vs. CPZ + RUT50 (ANOVA). NC: control group fed on normal diet; CPZ: cuprizone group fed on diet enriched with 0.2% (w/w) cuprizone; DMF: dimethyl fumarate 15 mg/kg; RUT50: rutin 50 mg/kg; RUT100: rutin 100 mg/kg; CC: corpus callosum; LFB/CV: luxol fast blue/cresyl-violet. Scale bar: 100 μ

High-intensity staining was obvious in the control group. The percentage area of myelination declined significantly in the CPZ group compared with the normal control (23.54 ± 1.093 vs. 44.42 ± 4.073; *p* < 0.01). Treatment with DMF, RUT50, and RUT100, however, attenuated this negative effect. In the RUT50 group, partial remyelination was observed (33.10 ± 0.739; *p* < 0.05). On the other hand, the remyelinated area was markedly increased in mice that underwent DMF and RUT100 therapy. They demonstrated LFB staining intensity comparable to the control group (39.77 ± 1.124, 42.98 ± 1.117, respectively; *p* < 0.01). Notably, there was a significant difference between RUT-treated groups, where the intensity of staining increased significantly in RUT100 compared to RUT50 group (*p* < 0.05; Fig. 3).

**Fig. 4:**
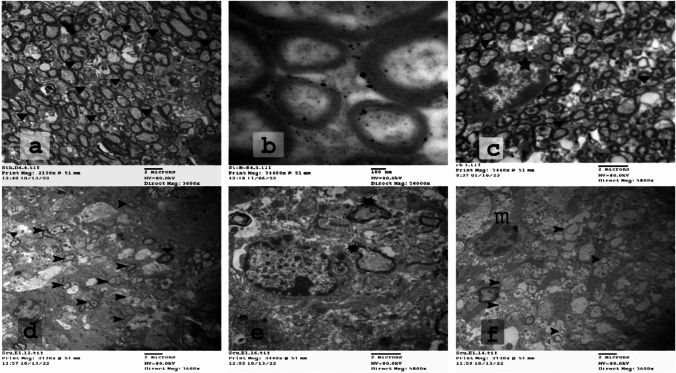
An electron micrograph cross-section of the CC from control (**a**–**c**) and CPZ-fed (**d**–**f**) groups after the sixth week of the experiment. **a** Large number of myelinated nerve fibers in the CC of normal mice. **b** Each fiber consists of an axon covered by a myelin sheath with consistent and parallel lamellae. **c** Oligodendrocytes ( ) were arranged between these myelinated fibers. In contrast, CPZ-fed mice **d** showed a few number of slightly myelinated axons ( ) and a higher number of unmyelinated axons (arrowhead ) after CPZ exposure. **e** The myelinated fibers showed injured myelin with many gaps between layers (asterisk ). **f** Abundant microglia were seen close to these fibers (m) (*n* = 3 per group). CPZ; cuprizone, CC corpus callosum

**Fig. 5:**
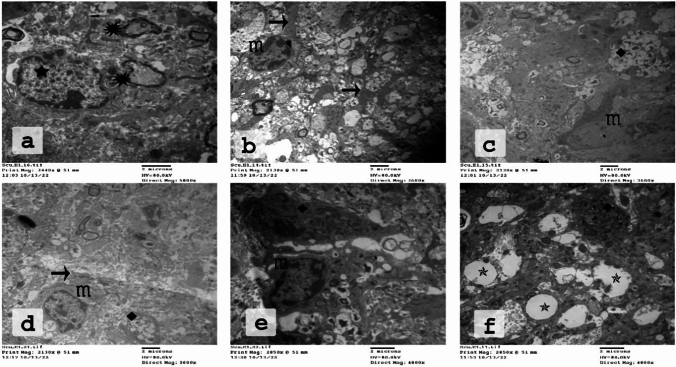
Electron micrographs of the CC of the CPZ-fed group showing stages of phagocytosis of myelin debris by microglia. Many distracted OLs were seen close to unhealthy nerve fibers. **a** Injured myelin ( ) and destructed OLs ( ) appear close to this injured myelin. **b**  Microglia spread their processes ( →) among these axons. **c**–**e** Phagosomes with many debris ( ). **f** Therefore, excessive demyelination and the accompanying phagocytosis might lead to numerous gaps and empty areas just next to microglia ( ). ( *n* = 3 per group). CPZ; cuprizone, CC corpus callosum; OLs: oligodendrocytes

**Fig. 6:**
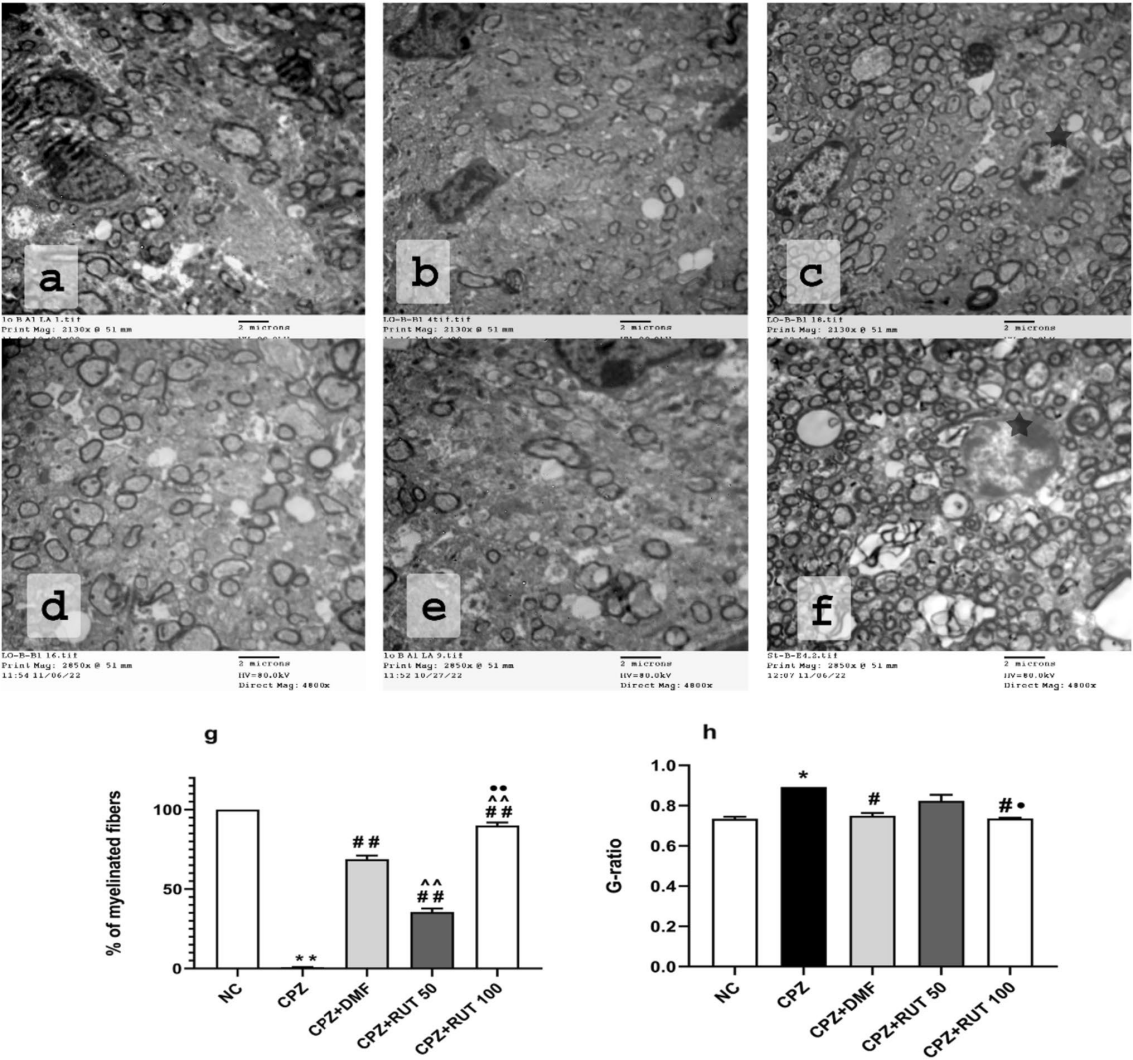
Myelination in different treatment protocols. In the DMF group (**a**, **d**), remyelination was demonstrated in many nerve fibers. However, in RUT50 (**b**, **e**), the myelinated axons were less than those in the DMF group. In RUT100 (**c**, **f**), the myelinated axons were more abundant, with a thicker myelin sheath, than both RUT50 and DMF. Oligodendrocyte cells ( ) were demonstrated frequently in the RUT100 group. **g** The percentage of myelinated fibers in the CC in TEM sections. **h** Quantification of the G ratio in the CC. Values are expressed as the mean ± SEM. * *p*  < 0.05, ** *p*  < 0.01 vs. NC group, ^#^ *p*  < 0.05, ^##^ *p*  < 0.01 vs. CPZ group, ^^^^ *p*  < 0.01 vs. CPZ + DMF, and ^•^ *p*  < 0.05, ^••^ *p*  < 0.01 vs. CPZ + RUT50 (ANOVA). NC: control group fed on normal diet; CPZ: cuprizone group fed on diet enriched with 0.2% (w/w) cuprizone; DMF: dimethyl fumarate 15 mg/kg; RUT50: rutin 50 mg/kg; RUT100: rutin 100 mg/kg; CC: corpus callosum

In addition to the examination of LFB-stained sections, we also performed an ultrastructural analysis and quantification for electron microscopic pictures. This is considered a much more accurate approach to determining the extent of myelination on the level of a single axon. Transmission electron microscopic photographs were used to determine the myelin morphometric parameters for axons from the CC of the control, CPZ, and treated groups (Figs. 4, 5, 6).

Results for the percentage of myelinated axons to the total axons are presented in Fig. 6g. In control conditions, we found thickly packed axons with a typical range in diameter and uniform regular myelin sheaths. In fact, control mice displayed healthy myelin sheaths, as shown by myelinated nerve fibers, which have an axon covered by myelin sheath with consistent and parallel lamellae. Their percentage was 100% (Figs. 4a–c, 6g). Cuprizone treatment resulted in a robust reduction in the myelinated axons. Only a few axons with normal myelin sheaths were hardly visible, where their percentage was 0.88% (*p*  < 0.01, Figs. 4d–f, 6g). The CC of mice that administered CPZ showed obvious demyelination as evidenced by axonal edema, flurry layers with gaps between them, as well as reduced electron density. In contrast, co-administration of DMF, RUT50, and RUT100 with CPZ prevented this decline in myelination, where the percentage of myelinated axons was 68.89, 35.56, and 90%, respectively (*p*  < 0.01, Fig. 6). The axons in the CC were nearly completely remyelinated after DMF and RUT100. As anticipated by the results of the behavioral tests and LFB staining, RUT100 group showed a remarkably significant increase in myelination compared to the standard drug as well as RUT50 (*p*  < 0.01). Additionally, the mean G-ratio showed a remarkable increase in the CPZ group compared with the control group (0.893 ± 0.00 vs. 0.735 ± 0.011; *p*  < 0.05). After DMF and RUT100 treatment, the G-ratio recorded an intermediate value (0.749 ± 0.014, 0.736 ± 0.004; *p*  < 0.05), while RUT50 produced an insignificant decrease in the G-ratio (0.824 ± 0.03) as compared to CPZ. The two doses of RUT differed significantly from one another (*p*  < 0.05), with a significantly lower G-ratio detected in the RUT100 group.

Shedding the light on the impact of CPZ intoxication on activation of microglia as shown in Fig. 5, we recognized OLs in the normal control group. Whereas after the administration of CPZ, we noticed the absence of OLs. We further noticed in CPZ-fed mice abundant microglial cells, which were hardly seen in the control group. These microglia engulfed many objects than usual. In some regions, we observed empty areas of different sizes with some enlarged mitochondria in between (Fig. 5f). Administration of the standard drug and RUT in the large dose along with CPZ significantly antagonized the CPZ-induced reduction in OLs and elevation in microglia, while RUT in the small dose produced insignificant effect (Fig. 6). These findings support our previous results showing that RUT treatment could enhance remyelinated axons in the CC area.

### Effect of rutin on cuprizone-induced inflammation

**Fig. 7:**
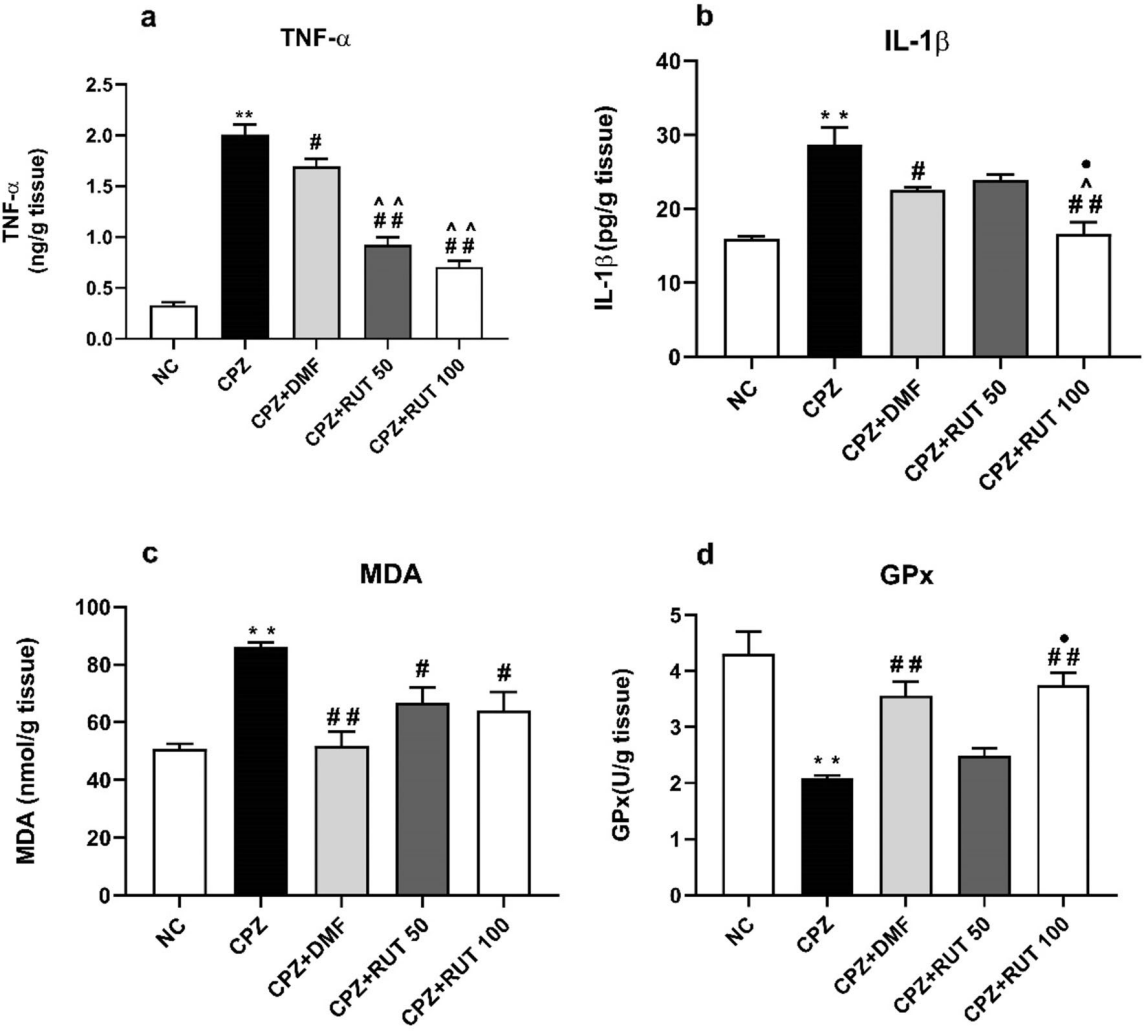
Effect of treatment protocols on the levels of TNF-α (**a**), IL-1β (**b**), MDA (**c**), and GPx activity (**d**). Data represent mean ± SEM. ** *p*  < 0.01 vs. NC group, ^#^ *p*  < 0.05, ^##^ *p*  < 0.01 vs. CPZ group, ^ *p*  < 0.05, ^^ *p*  < 0.01 vs. CPZ + DMF, and ^•^ *p*  < 0.05 vs. CPZ + RUT50 (ANOVA). NC: control group fed on normal diet; CPZ: cuprizone group fed on diet enriched with 0.2% (w/w) cuprizone; DMF: dimethyl fumarate 15 mg/kg; RUT50: rutin 50 mg/kg; RUT100: rutin 100 mg/kg; TNF-α: tumor necrosis factor-alpha, IL-1β: interlukin1-β, MDA: malondialdehyde, and GPx: glutathione peroxidase

As indicated in Fig. 7a and b, CPZ-treated mice demonstrated a dramatic increase in CC levels of TNF-α and IL-1β, compared to those that received normal chow (2.009 ± 0.092 ng/g tissue, 28.68 ± 2.324 pg/g tissue vs. 0.329 ± 0.0299 ng/g tissue, 15.97 ± 0.335 pg/g tissue; *p* < 0.01, respectively). Significant reduction in the elevated TNF-α levels was observed following 6 weeks of treatment with DMF, RUT50, and RUT100 (1.695 ± 0.072; *p* < 0.05, 0.924 ± 0.075, 0.707 ± 0.059 ng/g tissue, respectively; *p* < 0.01). Interestingly, RUT-treated mice at the two dose levels showed significantly lower levels of TNF-α than the standard drug (*p* < 0.01). Although in the RUT100 the TNF-α level was less than in the RUT50, no significant difference was detected between them. Similarly, IL-1β levels were markedly declined in the CC of animals treated with DMF and RUT100 (22.54 ± 0.358 for CPZ + DMF; *p* < 0.05, 16.60 ± 1.613 pg/g tissue for CPZ + RUT100; *p* < 0.01), compared to the CPZ group. On the other hand, RUT50 insignificantly reduced IL-1β level (23.93 ± 0.683 vs. 28.68 ± 2.324 pg/g tissue). Obviously, RUT100 produced a more pronounced reduction in IL-1β level than DMF and RUT50 (*p* < 0.05).

### Effect of rutin on cuprizone-induced oxidant/antioxidant markers

An imbalance between oxidants and antioxidants was observed in diseased mice in comparison to normal chow-fed ones. This was evidenced by a substantial rise in MDA level and a considerable lowering in the GPx activity (86.29 ± 1.528 vs. 50.88 ± 1.691 nmol/g tissue, 2.095 ± 0.045 vs. 4.307 ± 0.397 u/g tissue; *p* < 0.01, respectively). Conversely, the retrieval of oxidant/antioxidant status in the CC of RUT-treated mice was noticed by decreasing the lipid peroxidation marker and increasing the antioxidant enzyme activity. Rutin treatment significantly attenuated the elevation in the oxidative damage marker MDA in the CC of CPZ-fed mice (66.74 ± 5.450 for CPZ + RUT50, 64.21 ± 6.368 nmol/g tissue for CPZ + RUT100; *p* < 0.05). Notably, a remarkable difference was found between the two doses of RUT (*p*  < 0.05). Also, the GPx activity was considerably increased especially in the RUT100 group (3.753 ± 0.218; *p* < 0.01). Obviously, DMF greatly influenced the impaired oxidant/antioxidant balance observed in the CC of CPZ-fed mice (51.97 ± 4.735 vs. 86.29 ± 1.528 nmol/g tissue for MDA, 3.565 ± 0.241 vs. 2.095 ± 0.045 u/g tissue for GPx; *p* < 0.01; Fig. 4c, d).

### Molecular modeling

Modeling and docking simulations were performed to ascertain the mechanistic action for the anti-inflammatory and antioxidant effects of RUT using Molecular Operating Environment (MOE) software https://www.chemcomp.com/. Initially, the present study aims at determining whether RUT has an inhibitory binding affinity of NF-κB to investigate its anti-inflammatory effect. The docking protocol was done using the crystal structure of human NF-κB P50 homodimer bound to DNA (PDB: 1SVC) (Müller et al. 1995).

**Fig. 8:**
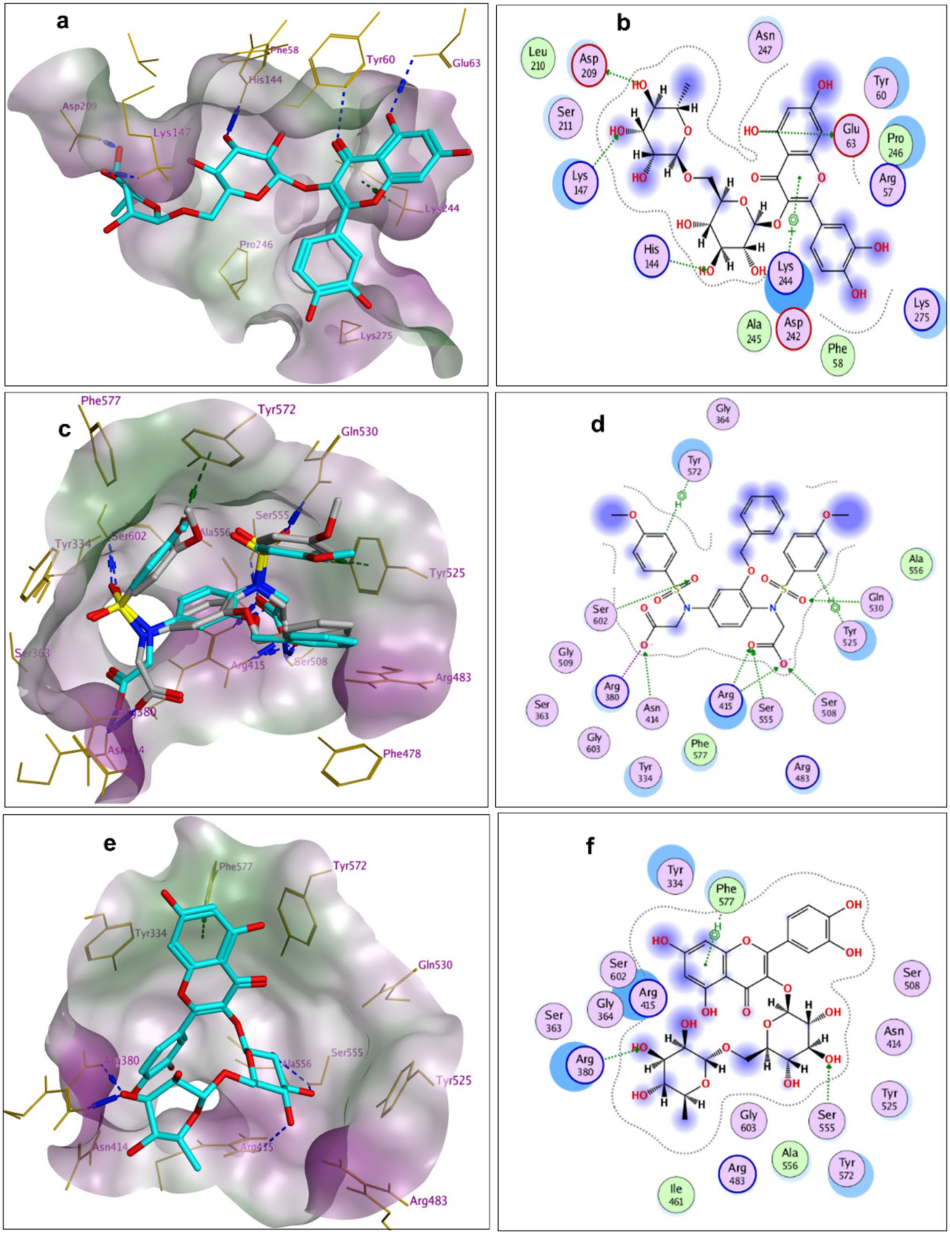
3D and 2D docking representations of protein–ligand complexes; **a** 3D-docked model of RUT (cyan) showing the lipophilicity surface of NF-κB active site; (hydrophilic; green: hydrophobic, purple; neutral; white); **b** 2D-docked model of RUT within NF-κB active site; **c** 3D-docked model of redocked ligand (cyan) aligned with the co-crystallized ligand (grey) showing the lipophilicity surface of Keap1 active site for the Kelch domain (hydrophilic; green: hydrophobic, purple; neutral; white); **d** 2D-docked model of redocked ligand within Keap1 active site for the Kelch domain; **e** 3D docked model of RUT (cyan) showing the lipophilicity surface of Keap1 active site for the Kelch domain (hydrophilic; green: hydrophobic, purple; neutral; white); **f** 2D-docked model of RUT within the active site of Keap1 for the Kelch domain

A structural and active site prediction of NF-κB was performed by means of computed atlas of surface topography of proteins (CASTp) (Tai et al. 2020), as well as MOE Site Finder tool (MOE, 2019.01). Results revealed that RUT binds snugly at the NF-κB binding pocket of protein-DNA complex with a docking *score* of −6.79 kcal/mol forming several interactions with the surrounding amino acid residues (Fig. 8a, b). The ligand aglycone stacks between Tyr60 and His144 forming two hydrogen bond interactions with Glu63 and Tyr60 (3.14 Å) along with a pi-H interaction with Lys244 (3.62 Å). In addition, the sugar moiety forms multiple H-bond interactions with His 144, Asp209, and Lys147 with (3.12, 2.88 and 3.12 Å, respectively). Other ligands interactions within the active site include hydrophobic ones with Tyr60, Pro246, Lys275, Lys244, Lys147, and Asp209.

Additionally, RUT was further subjected to a docking study within the active pocket of human kelch-like ECH-associated protein1 (Keap1) for the Kelch domain; (PDB: 6HWS) https://www.rcsb.org/structure/6HWS to predict the mechanistic action of its antioxidant effect. The docking protocol was validated by redocking the co-crystallized ligand that exhibit S score of −11.7868 kcal/mol with RMSD value of 0.8893 Å (Fig. 8c, d). Docking results of RUT within the protein active site revealed that the compound exhibited a good negative docking score of −8.6148 kcal/mol as well as forming some interactions with the nearby amino acid residues compared with the co-crystallized inhibitor (Fig. 8e, f). The ligand aglycone stacks between Tyr572 and Tyr334 forming pi-hydrogen interaction with Phe577 (3.82 Å). Moreover, the sugar moiety forms H-bond interactions with Ser555 (2.99 Å) and engages two hydrogen bonds with Arg380 (3.30 and 2.95 Å). In addition, other ligands interactions within the active site include hydrophobic ones with Tyr572, Phe577, Tyr334, Arg415, and Arg380. Results suggested that RUT might act by inhibiting Keap1–Nrf2 protein–protein interaction and allow activation of the Nrf2-ARE signaling pathway for the antioxidant effect.

### Effect of rutin on cuprizone-  induced alterations in Nrf2 expression

**Fig. 9:**
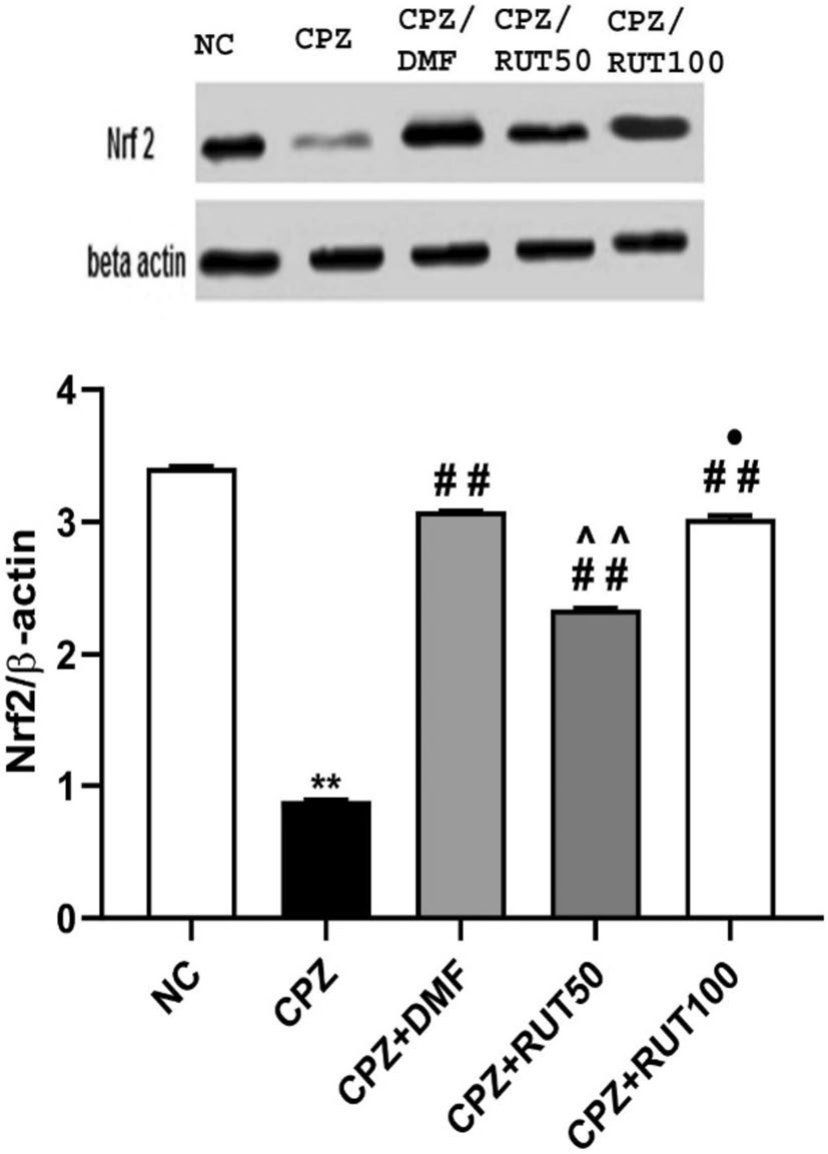
Effect of treatment protocols on Nrf2 expression. Data represent mean ± SEM. ** *p*  < 0.01 vs. NC group, ^##^ *p*  < 0.01 vs. CPZ group, ^^^^ *p*  < 0.01 vs. CPZ + DMF, and.^•^ *p* < 0.05 vs. CPZ + RUT50 (ANOVA). NC: control group fed on normal diet; CPZ: cuprizone group fed on diet enriched with 0.2% (w/w) cuprizone; DMF: dimethyl fumarate 15 mg/kg; RUT50: rutin 50 mg/kg; RUT100: rutin 100 mg/kg; Nrf2: the transcription factor (erythroid-derived) nuclear factor-like 2

Cuprizone intoxication induced 74% decrease in Nrf2 protein expression. However, treatment with DMF, RUT50, and RUT100 significantly attenuated the CPZ-induced reduction in Nrf2 expression, and restored the level to 90, 68 and 89%, respectively (Fig. 9).

## Discussion

Instead of providing a thorough coverage of many aspects of the pathophysiology underlying the disease or supporting the recovery of the injured tissues, most of the current MS therapy regimens concentrate on suppressing the immune response (Coles et al. 2022; Filippi et al. 2022). These medications have considerable prices and adverse safety profiles, and they are only modestly effective at treating MS, with variable efficacy in targeting the degenerative components of the disease. Consequently, there is a constant need for new medications with improved safety profiles and novel mechanisms of action.

Accumulating evidence suggests that oxidative stress and inflammation contribute to the etiology of MS. Numerous biological and pharmaceutical effects of RUT include anti-inflammatory, antioxidant, antihypertensive, anti-apoptotic, and anticancer effects (Perk et al. 2014). Additionally, RUT was reported to cross the BBB (Anjomshoa et al. 2020). Yet, to our knowledge, no reports have so far indicated the potential benefit of RUT in MS. Consequently, the present study was designed to examine the neuroprotective effects and possible mechanisms of action of the natural flavonoid; rutin (3,3,4,5,7-pentahydroxyflavone-3-rhamnoglucoside), against CPZ-induced MS in mice, in comparison to a conventional drug; DMF. The coworkers of the present study focused on evaluating several parameters, including body weight, behavior,  remyelination by histopathology and TEM, interaction with target proteins by molecular modeling,  as well as biochemical analyses for oxidative stress and inflammatory biomarkers.

Considering the complex pathophysiology of MS, there are currently no experimental animal models that fully represent the disease. Instead, a variety of experimental models and intoxications can be used to focus research and identify specific pathogenic pathway and MS healing mechanisms (Kipp et al. 2009; El Sharouny et al. 2021). Indeed, the CPZ-fed mice model shows subtle cognitive and locomotor deficits. Oral administration of this copper chelator was reported to cause demyelination in distinct brain regions, particularly the CC; the extensively investigated white matter tract in this animal model. Animals of this model do not undergo severe motor deficits, such as paralysis, due to limited pathology of the spinal cord, and so is quite similar to early human MS motor symptomology (Sen et al. 2022).

In MS patients with pattern III lesions, demyelination is caused by a functional disturbance of OLs, possibly as a consequence of infection or damage interceded by some unknown toxins (Kipp et al. 2009). According to a number of researches, CPZ demyelination model is similar to the type III lesion histological pattern (Torkildsen et al. 2008; Zirngibl et al. 2022). When CPZ is fed to animals, it results in a predictable and repeatable pattern of non-autoimmune-mediated demyelination (Praet et al. 2014; Zhan et al. 2020). It causes cell death of the OLs, which are the sole cells responsible for myelination in the CNS, leading to subsequent demyelination (Luo et al. 2021). Oligodendrocytes are the cells specifically susceptible to changes in energy metabolism and oxidative imbalance owing to their high level of metabolism (Benetti et al. 2010; Nellessen et al. 2020). As CPZ is a copper chelator and inhibits copper-dependent enzymes such as superoxide dismutase (SOD), it leads to mitochondrial dysfunction, oxidative stress, selective OLs apoptosis, myelin sheath degeneration, axonal beading, microgliosis, astrogliosis, and neuroinflammation similar to that found in MS (Sen et al. 2022). Hence, this model was selected in the present study to examine the possible impact of antioxidant and anti-inflammatory therapies in remyelination and amelioration of MS.

In the current study, we reconfirm that CPZ treatment resulted in substantial systemic neurotoxicity in the tested mice, characterized by body weight loss, motor dysfunction, demyelination, and glial activation in demyelinated lesions. Furthermore, we demonstrated that CPZ resulted in significant oxidative stress and proinflammatory cytokines elevation. These findings are matching with other reports (Aryanpour et al. 2017; Madadi et al. 2022). An important point is that spontaneous remyelination was reported to occur 4 days after the neurotoxin has been removed (Lindner et al. 2008). Therefore, the administration of CPZ was continued all over the 6-week period of the study.

The selection of DMF, as a standard MS drug in our study, was based on its efficiency as an Nrf2 activator, resulting in amelioration of oxidative stress, neuroinflammation, OLs loss, and axonal damage (Gold et al. 2012; Nellessen et al. 2020). Most drugs approved in MS therapy act by immunosuppression, which may not be representative standards to the CPZ model; a non-autoimmune MS model relying mainly on primary apoptosis of OLs, oxidative stress, and neuroinflammation to induce the brain lesion.

In the current work, we investigated the potential of RUT as an approach to reversing CPZ-induced neurotoxicity. The previously reported weight loss induced by CPZ (Hashimoto et al. 2017; Almuslehi et al. 2020) was also detected in our study, this may be due to a decrease in food consumption as well as an increase in basal metabolic rate (Omotoso et al. 2018). Mice treated with DMF or RUT, simultaneously with CPZ, showed a linear increase in body weight. The final weight of mice treated with the higher dose of RUT was close to that of the control group, which indicated that RUT counteracted CPZ-induced weight loss. Similar effect of RUT in restoring body weight  has been reported by Suganya and Sumathi (2017). These authors stated that no considerable alterations in body weight were observed in animals treated by RUT alone as compared to the control. However, RUT in that study significantly prevented the loss of body weight in Huntington’s diseased-rats. The potential mechanism by which RUT maintains body weight might be its ability to increase food intake (Parashar et al. 2017). In contrast, Seo et al. (2015) reported that RUT consumption decreased body weight compared to the high-fat diet group. The controversy of these results to ours may be attributed to difference in dietary types, animal models or species, experiment duration, or RUT dose.

Animal studies on the CPZ model reported that demyelination and behavioral impairment co-occurred in mice brains (Faizi et al. 2016). Our results from open field, rotarod, actophotometer, and string grip strength tests showed that CPZ ingestion resulted in behavioral and motor deficits, as compared to the control group. Behavioral dysfunction in CPZ-treated animals was first reported by Morell et al. (1998) and has been replicated with standardized tests (Torkildsen et al. 2008). Treatment with DMF or RUT significantly improved behavioral and motor abnormalities, where mice were more active and balanced, suggesting the possible amelioration of CPZ-induced demyelination. These findings were coincidence with those of Suganya and Sumathi (2017), who reported that RUT treatment significantly restored behavioral alterations in an experimental model of Huntington’s disease.

One of the main characteristics of MS is the loss of the myelin sheath. The area percentage of demyelination in the CC in LFB/CV-staining provides a good parameter to study myelin sheath. In the present study, CPZ showed a significant decrease in the percentage area of LFB/CV-staining characterized by light or low staining density in CC, which is in agreement with previous reports (Acs et al. 2009; Sághy et al. 2016). However, in DMF or RUT-treated groups, and particularly RUT100, the degree of LFB staining intensity was significantly higher than that in the CPZ group, demonstrating their ability to prevent demyelination or to initiate remyelination.

The TEM was performed to further confirm the protective effect of RUT against demyelination. The CC of mice that received CPZ showed obvious demyelination as evidenced by axonal edema, flurry layers with gaps between them, as well as reduced electron density, which is in harmony with previous studies (Lindner et al. 2008; Tahmasebi et al. 2021). Axonal diameter, myelin sheath, and the number of myelinated axons were all dramatically reduced by CPZ, consequently resulting in increased G-ratio (Tahmasebi et al. 2021). On the other hand, administration of RUT or DMF to CPZ-fed mice preserved intact morphology. Structurally, this improvement was demonstrated by the appearance of an abundance of myelinated nerve fibers in the CC, and this number was remarkably higher with the large dose of RUT followed by the standard drug. In addition, RUT100 and DMF increased the diameter and thickness of myelin fiber, and consequently decreased the G-ratio. Again, RUT100 produced the most pronounced reduction in G-ratio, which was close to that of the control group.

Reactivation of astrocytes and microglia, together with their accumulation, mitochondrial dysfunction, and release of ROS are major contributors to OLs apoptosis and demyelination (Zirngibl et al. 2022). Thus, approaches that modify these mechanisms may potentially provide a new range of therapeutic strategies. Based on the well-known RUT-mediated cytoprotection against oxidative stress and inflammation, we estimated its potential protective effect in the CC of CPZ-fed mice.

In the current study, elevation of MDA concentrations, together with a decrease in GPx activity, were detected in the CC of CPZ-fed mice. There is numerous evidence that accused oxidative stress in the development of MS. Cuprizone-induced copper deficit is harmful to mitochondrial function, disturbing the antioxidant system, and elevating ROS in OLs leading to apoptosis (Largani et al. 2019). On the contrary, treatment with DMF and the high dose of RUT was able to restore GPx activity and MDA concentration within the range of control. Our results were consistent with earlier research emphasizing the central antioxidant effect of RUT in various animal models such as Cisplatin-induced neurotoxic rat model and fluoride-treated rats (Almutairi et al. 2017; Nkpaa and Onyeso 2018). In harmony, results of the molecular modeling threw some light on the possible mechanism by which RUT might exert its beneficial antioxidant effects that protected against CPZ-induced demyelination. Indeed, RUT inhibited the Keap1–Nrf2 protein–protein interaction and allowed activation of the Nrf2-ARE signaling. Furthermore, our data by western blot demonstrated that DMF, and   RUT100  significantly upregulated the expression of Nrf2 more than RUT50. Taken together, results of molecular modeling and western blot analysis strengthen each other, and support the impact of RUT in modulating Nrf2 pathway, and the possibility that RUT could represent a novel approach in MS treatment.

Neuroinflammation is another well-known feature of MS (Bernal-Chico et al. 2020; Beckmann et al. 2023). Importantly, TNF-α and IL-1β, generated by activated microglia and astrocytes, are powerful effectors of OLs death, demyelination, and axonal damage (Zirngibl et al. 2022). Our findings showed elevated levels of TNF-α and IL-1β in the CC of CPZ-fed mice, providing evidence of neuroinflammation induced by CPZ ingestion, as formerly reported (Liu et al. 2018; Khalilian et al. 2021). In contrast, RUT in large dose mitigated neuroinflammation by significantly reducing TNF-α and IL-1β levels, which is consistent with previous reports on RUT in neurodegenerative diseases including Alzheimer’s disease, Parkinson’s disease, and Huntington’s disease (da Silva et al. 2017; Enogieru et al. 2018). A possible mechanism underlying this effect might be the inhibition of NF-κB; one of the principal transcription factors involved in the control of inflammation and the expression of a wide range of inflammatory mediators. This was confirmed by a molecular modeling study, where RUT binds snugly at the NF-κB binding pocket of protein-DNA complex and exhibited an inhibitory binding affinity to NF-κB. Interestingly, the anti-inflammatory effect of the standard drug was much less than that of RUT. One possible explanation might be that DMF produces its effects by acting mainly as an Nrf-2 activator (Nellessen et al. 2020). As neuroinflammation is a key contributor to the pathophysiology of MS,   a powerful anti-inflammatory agent, RUT, could emerge as a candidate for treatment of MS. Also, the combined antioxidant and anti-inflammatory activities could represent superiority of RUT over the conventional drug; DMF.

The human leukocyte antigen (HLA) super locus on chromosome 6p21, which consists of Class I, II, and III, is largely acknowledged as having the greatest influence on the aetiology of  MS  among the genetic risk factors and  is crucial in adaptive immune reactions. In addition to their physiological function in protecting the host from infectious microorganisms, several alleles operate as genetic risk factors for autoimmune disorders like MS. HLA exerts the largest genetic contribution to MS susceptibility (Alcina et al. 2012). HLA system or complex encodes cell-surface proteins responsible for the regulation of the immune system. The HLA system is also known as the human version of the major histocompatibility complex (MHC) found in many animals (Heijmans et al. 2020). Immune cells residing in the CNS get activated following damage to CNS tissue; notably microglial cells whereby they upregulate MHC class I and II molecules and cell surface co-stimulatory molecules secrete cytokines and chemokines, paving entry for T (CD4 and CD8) cells, B cells, monocytes, macrophages and dendritic (DC)-like cells into CNS lesions. Infiltrating immune cells secrete pro-inflammatory cytokines, nitric oxide, and matrix metalloproteinases, leading to the destruction of the myelin sheath (Dargahi et al. 2017). There are many factors that influence HLA expression in both resting and activated cells. HLA is expressed with specific promoter proteins such as CIITA (Carey et al. 2019). The rise in the IFN-γ is accompanied by the upregulation of CIITA expression which in turn promoted the expression of MHC-II molecules that are responsible for the binding of CD4 + T cells (Ng et al. 2021). Data revealed that flavonoids inhibit antigen-specific memory T cell proliferation and pro-inflammatory IFN-γ production (Verbeek et al. 2005). For example, the treatment of the *P*. *berghei*-infected mouse model with RUT at lower doses (25 and 50 mg/kg) showed a significant reduction in the secretion of pro-inflammatory cytokines (TNF-α, IL-6 and IFN-γ) and accompanied by decrease of CIITA expression then HLA expression (Bhatt et al. 2022). Moreover, NF-κB pathway certainly contribute to MHC class I expression via the NF-κB binding Enhancer A region and interferon (IFN)-sensitive response element (ISRE) motif in the MHC class I promotor region (Jongsma et al. 2019). Our results showed that RUT had an inhibitory effect to NF-κB, and therefore, we suggest by this way that RUT might affect HLA gene.

Overall, findings of the present study suggest that RUT could promote remyelination in CPZ-intoxicated mice via increasing antioxidant enzymes, along with decreasing oxidative stress  and inflammatory cytokines, which are hallmarks of MS. This was evident by its ability to restore body weight, improve motor coordination and balance, increase the intensity of LFB staining, and increase the number of myelinated axons and thickness of myelin. These obvious valuable effects of RUT on CPZ-induced demyelination, which might be mediated by inhibition of NF-κB and Keap1–Nrf2 protein–protein interaction, in addition to its possibility to affect HLA gene, suggest that RUT could emerge as a candidate for MS treatment.

## Conclusion

In this study, we investigated the neuroprotective effects of RUT on demyelination induced by CPZ. Cuprizone significantly reduced body weight and impaired muscle coordination and locomotion. The histopathological study showed that the CC has severely lost myelinated fibers. Moreover, CPZ increased brain inflammation and oxidative stress. Oral administration of RUT100 for 6 weeks was effective in alleviating all these sequelae, and RUT effect was obviously more marked than that of the standard drug regarding most of the tested parameters. Additionally, based on the behavioral, histological, and biochemical studies, 100 mg/kg of RUT was obviously more effective than RUT50 mg/kg. These results are one of the first to imply that RUT100 may successfully improve remyelination. The antioxidant and anti-inflammatory properties of RUT in brain tissue are the most likely mechanisms causing these protective effects. These beneficial properties may be mediated by modulating NF-κB, and Nrf2-ARE signaling pathways. This research may also open the door to investigating additional mechanisms that underlie RUT neuroprotective benefits. Results of the current study reinforce the use of large doses of RUT in the treatment of demyelinating illnesses like MS as a potential candidate to alleviate symptoms that may be partially caused by demyelination as a result of oxidative stress and inflammation. Taking into account its natural source, as well as its potential higher safety profile, RUT could emerge as a useful and safe therapeutic alternative, or even an adjuvant, to synthetic drugs approved in MS treatment, with a possible disease-modifying effect that could favorably influence disease progression.

## Data Availability

Any required additional data that support the findings of the present study will be available from the corresponding author upon request. Some data may not be publicly available due to restrictions that could compromise the privacy of research participants.

## Abbreviations

CPZ: Cuprizone
OLs: Oligodendrocytes
NF-κB: Nuclear factor κB
MDA: Malondialdehyde
GPx: Glutathione peroxidase
TNF-α: Tumor necrosis factor-alpha
IL-1β: Interleukin-1β
IFN-γ: Interferon-γ
DMF: Dimethyl fumarate
RUT50: Rutin (50 mg/kg; p.o)
RUT100: Rutin (100 mg/kg; p.o)
OFT: Open-field test
TEM: Transmission electron microscope
LFB: Luxol fast blue
CV: Cresyl-violet
Keap1: Kelch-like ECH-associated protein 1
Nrf2: Transcription factor (erythroid-derived) nuclear factor-like 2

## Declarations

### Author agreement

The authors warrant that this manuscript is original, has not been formerly published and is not currently under consideration for publication by another journal. We authorize that the manuscript has been revised and approved by all authors and that all persons who satisfied the criteria for authorship are listed. The authors also agree to the order of authors listed in the manuscript, and to the sole responsibility of the corresponding author for contacting the Editorial Office, and communicating with other authors about progress, submissions of revisions, and final approval of proofs.
